# Apocynum Cannabinum in Dropsy

**Published:** 1881-03

**Authors:** 


APOCYNUM CANNABINUM IN DROPSY.—Dr. T. S. Dabney, in an interesting and instructive paper read before the New Orleans Medical and Surgical Association, revives the use of this remedy in dropsy, Bright's disease and other chronic affections of the kidneys, liver and heart, giving rise to dropsical effusion, which were all benefited by its diuretic and tonic properties. He used a tincture. " One ounce of the fresh root makes six ounces of strong tincture. Of this the dose is from gtt. v. to gtt xxx."

